# Predicting individual hemoglobin abnormalities using longitudinal data in clinical practice

**DOI:** 10.1186/s12911-025-03085-6

**Published:** 2025-07-15

**Authors:** Maliheh Namazkhan, Karel Jan van Tuijn, Maurits Kaptein, Remco van Horssen

**Affiliations:** 1 Department of Methodology and Statistics, Tilburg School of Social and Behavioral Sciences, University of Tilburg, Tilburg, The Netherlands; 2 Health Center Pannenhoef, General Practitioner, Kaatsheuvel, The Netherlands; 3 Department of Mathematics and Computer Science, Eindhoven University of Technology, Eindhoven, The Netherlands; 4 Department of Clinical Chemistry and Hematology, Elisabeth-Tweesteden Hospital, Tilburg, The Netherlands

**Keywords:** Individual hemoglobin value, Prediction model, Longitudinal data, Generalised additive model, Preventive health

## Abstract

**Background:**

In preventive medicine, the promotion of health and well-being through early detection and intervention is crucial to preventing the development of diseases. This study aims to predict potential abnormalities in hemoglobin levels before they occur, using individualised observations within normal ranges.

**Methods:**

We utilise a dataset generated over seven years, comprising 30,000 patients. Multiple prediction models are employed to identify hemoglobin trends within individuals and predict their next-to-measure hemoglobin value based on past measurements. We focus on whether, at a specific point in time, the individual’s values are likely to run outside of the individual ‘normal’ bounds. A Generalised Additive Model is explored as a plausible approach for predicting future individual hemoglobin values. By calculating confidence intervals for predicted hemoglobin values, we evaluate prediction uncertainty, while assessing the percentage of accurate predictions within these intervals to gauge the reliability of our model’s prediction.

**Results:**

We find that for 88.47% of the cases, our model accurately predicts whether patients’ hemoglobin levels will stay within individual ‘normal’ bounds or deviate from them, demonstrating its effectiveness in identifying ‘out-of-normal’ measurements.

**Conclusions:**

The findings hold practical significance, potentially reducing unnecessary blood draws and preventing the onset of abnormal hemoglobin levels through preventive healthcare interventions or digital lifestyle coaching. Moreover, early detection and intervention can significantly impact individual patients by preventing disease development.

## Background

Preventive medicine aims to promote health and well-being by proactively identifying and addressing potential health issues before they become diseases [1, 2]. A key aspect of preventive medicine is personalised or precision medicine, which promotes healthcare interventions to individual characteristics, such as genetic profile, environmental exposures, and lifestyle factors [3, 4].

Personalised medicine has gained significant traction in recent years, driven by advancements in technologies like genomics, proteomics, and digital health monitoring [5–7]. These technologies have revealed substantial inter-individual variation in disease processes, highlighting the need for personalised approaches to diagnosis, treatment, and prevention [5, 8].

Despite these advancements, routine laboratory medicine still predominantly relies on reference ranges for blood parameters derived from population studies, which often fail to account for individual variation. For instance, the normal range for hemoglobin (Hb), a crucial marker for anemia, is generally defined as 7.5–10.0 mmol/L for women [9]. However, individual Hb values can vary significantly within this range due to biological variability between individuals [10]. For example, an Hb value of 7.8 mmol/L might be considered normal based on population-based reference ranges, but it could be unexpected for a specific individual if their previous measurements consistently remained around 9.5 mmol/L. Both these values fall within the normal range for adults [11, 12]. However, this kind of variation highlights the importance of considering individualised baselines rather than relying solely on population-based reference ranges, as the latter might overlook clinically relevant deviations in an individual context [13].

The estimation of individual Hb levels by deep learning models is of specific interest, as Hb is a very relevant blood parameter in medical care [14]. By using individualised observations, it may be possible to anticipate potential abnormalities in Hb levels before they become clinically significant, thus preventing unnecessary blood draws and costly hospital visits. This approach aligns with the principles of personalised medicine and could significantly impact individual patients by facilitating early intervention and preventing disease development [3, 7]. Furthermore, recent research has shown that blood test results are highly individual, with stable personalised ranges, suggesting the potential for more precise diagnostics [15].

In this study, we explore the use of Generalised Additive Models (GAMs; [16]) for predicting individual Hb levels. The inherent flexibility of GAMs in capturing the complex, non-linear relationships between predictor variables and response variables suggests their potential applicability for predicting individual Hb trends over time. GAMs are specifically designed for analysing longitudinal data with dynamic patterns. GAMs flexibility in accommodating time-dependent covariates and incorporating smoothing functions allows for accurate modelling of individual Hb trajectories. Furthermore, GAMs provide interpretable results, facilitating the visualisation of Hb trends and aiding in the identification of clinically relevant patterns. These attributes make GAMs particularly suitable for predicting individual health trajectories over time.

This flexibility is a major advantage over other statistical tools such as mixed effects models, which are often limited in capturing complex non-linear patterns and interactions between variables. In contrast, GAMs are capable of fitting non-linear relationships with smooth functions, allowing for a more comprehensive insight into the temporal dynamics of Hb levels. Moreover, unlike mixed effects models, GAMs can incorporate sophisticated smooth functions that capture both individual variability and time-dependent effects. In our data, this capability is particularly advantageous, as we observe significant non-linear trends in Hb levels over time that are better captured by GAMs. Thus, GAMs provide a more precise interpretation of individual Hb trends, which aligns closely with the goals of our analysis.

Yet, a critical research gap exists in developing predictive models that can effectively predict individual Hb values based on previous measurements within normal ranges. While recent studies have explored various machine learning approaches to estimate or predict Hb-related parameters [14, 17, 18], there is a lack of research focused on individual-level analysis and explicit reference to personalised thresholds. Our study addresses this gap by using GAMs to model individual Hb trajectories over time and develop personalised predictions and thresholds. Rather than relying on population-based reference ranges, this approach emphasises the importance of personalised medical interventions based on individual data and trends. We specifically target patients whose Hb values are anticipated to remain within personalised normal ranges. Notably, our model does not incorporate clinical data. The novelty lies in the GAMs application to capture individual variations in Hb levels and facilitate a shift from population-level to individual-level analysis in preventive healthcare.

While we can effectively model individual variations and develop personalised predictions, we lack a fully developed method to monitor deviations from these individualised thresholds over time. However, we recognise the importance of such a monitoring system and propose it as a potential area for future research. In this study, we aim to investigate the feasibility and effectiveness of using GAMs to model individual Hb trajectories and predict future Hb values based on historical data of non-hospitalised individual primary care patients whose Hb values were measured in the laboratory of the Elisabeth-Tweesteden Hospital (ETZ) in the Netherlands. Specifically, we examine the predictive performance of GAMs for anticipating individual Hb trends and preventing unnecessary blood draws and costly hospital visits.

## Method

### Data collection

We accessed Hb data from a cohort of 118,270 unique primary care patients spanning the period from 2012 to 2019. Regular Hb checks are typically conducted as part of routine health monitoring for chronic diseases or due to specific medical conditions requiring frequent monitoring. Our study focused on a subset of 110,657 adult patients aged 16 and above. To ensure the inclusion of patients with predominantly stable Hb levels within a normal range throughout the study period, we applied stringent inclusion criteria. Specifically, we excluded 54,204 participants whose Hb values fell outside the reference range (Hb less than 6 mmol/L or above 16.5 mmol/L) and those with fewer than 3 or more than 100 Hb measurements. This selection aimed to capture patients who likely underwent regular Hb checks as part of standard medical care. Our final sample included 56,453 patients, consisting of 33,432 females and 23,021 males. Table 1 presents a summary of demographic characteristics of patients, including mean age and gender distribution considered in our analysis.

**Table 1:** Descriptive for demographic variables

Variable	Response categories
Patient age	M^1^ = 65.79, SD^2^ = 18.34, Min = 17, Max = 119
Patient gender	Female (59.23%), Male (40.77%)

^1^ M = Mean

^2^ SD = Standard Deviation

### Hb measurement

The data consists of multiple measurements of patients’ Hb values recorded over seven years (2012–2019). In short, the Hb, the primary component of red blood cells, plays a critical role in oxygen transport throughout the body. The number of Hb measurements per patient ranged from 3 to 100, with a median of 13 and a standard deviation of 16.34. Table 2 presents the reference values for Hb, describing normal ranges by gender.

**Table 2:** Hemoglobin actual value, reference range among gender

Parameter	Actual value	Reference range	Units	CVa^1^(%)	TEa^2^(%)
Hemoglobin (Hb)	M = 8.09, SD = 1.04 Min = 6.10, Max = 14.30	Male: 8.5–11.0 Female: 7.5–10.0	mmol/L	1.43	4.19

^1^ CVa: analytical variation/imprecision, a measure of the reproducibility performance of a test [23]

^2^ TEa: Total Allowable Error, a measure of the accuracy of a test defined as the acceptable difference between the measured and actual value [23]

**Fig. 1:**
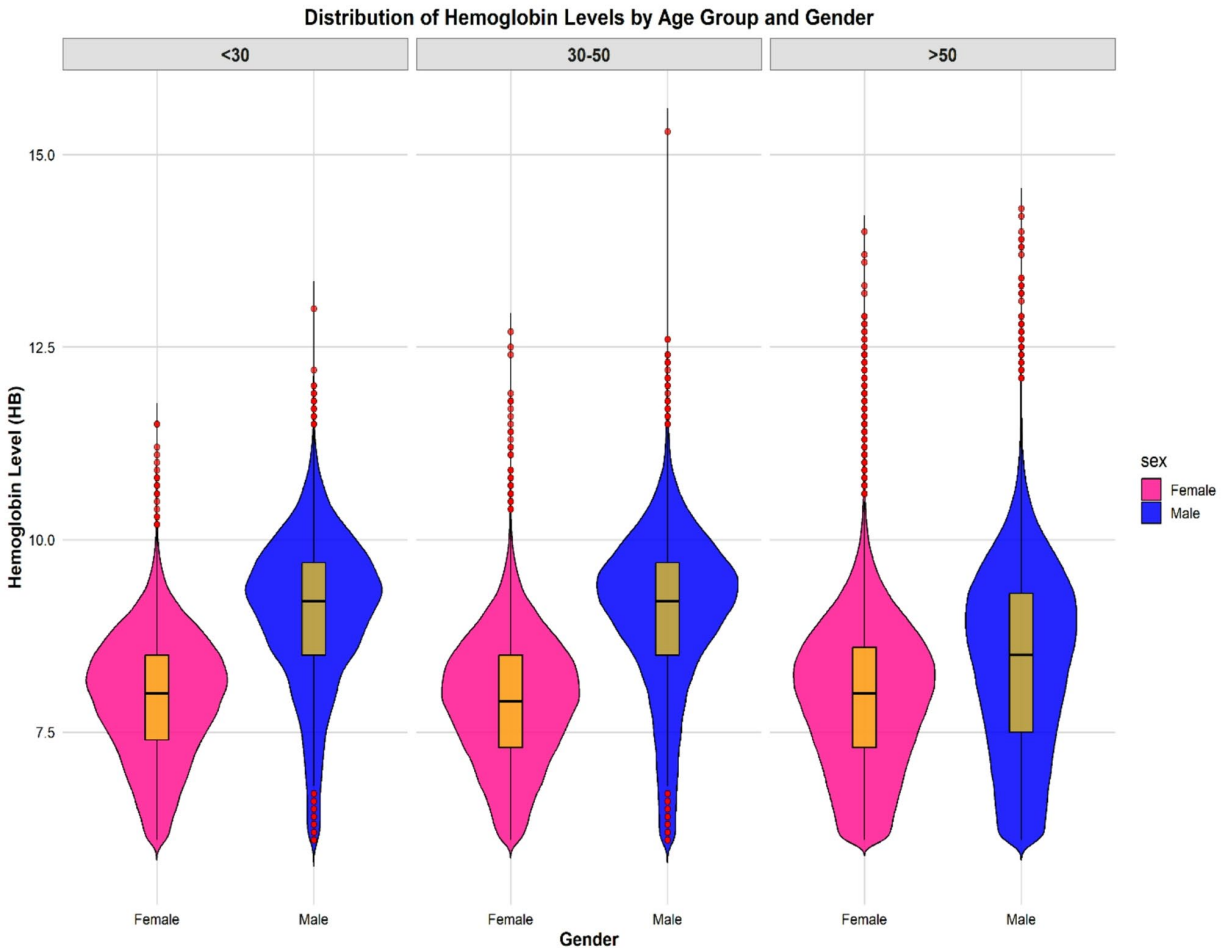
The figure displays violin plots with boxplots of Hb levels across age groups, categorised by gender for the period between 2012 and 2019 (*n* = 56,453). The x-axis represents the age groups (< 30, 30–50, and > 50), while the violin plots are separated by gender. Pink represents females, while blue represents males, clearly depicting the differences in Hb distribution between genders. Each panel represents an age group, illustrating how the Hb level distribution differs across genders and age groups. Within each violin plot, the width represents the density of Hb values for each gender in each age group. The boxplots are coloured in yellow and show key statistical summaries: the thick black line indicates the median Hb level, while the box represents the interquartile range. Thin lines extending from the box indicate the range within which the Hb levels fall, providing insight into the maximum and minimum values observed Red points identify outliers, and highlight data points that lie beyond 1.5 times the interquartile range from either end of the box

**Fig. 2:**
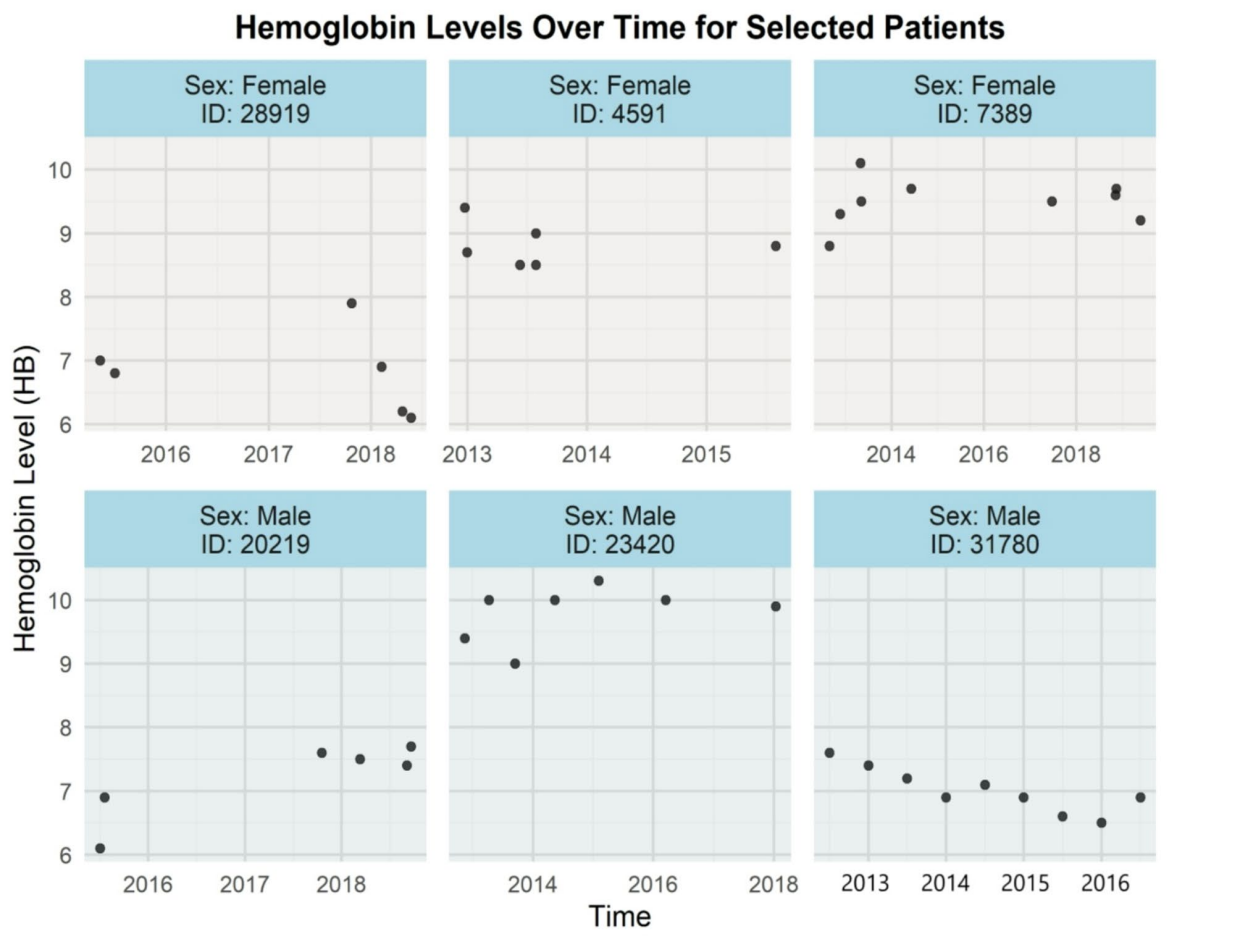
This figure displays Hb values for six selected patients over the period 2012–2019, with each panel representing the patient’s sex and ID. The black dots denote individual Hb measurements over time for each patient, allowing for a clear visualisation of the distinct patterns and variations in Hb levels for each individual

Additionally, Fig. 1 displays violin plots overlaid with boxplots showing Hb levels across age groups (less than 30 years, 30 to 50 years, and greater than 50 years), categorised by gender, from 2012 to 2019. The Hb level distributions reveal that males generally have higher median Hb levels compared to females, with variability increasing with age for both genders. These trends are consistent with existing literature, which suggests that both age and gender significantly influence Hb concentrations. Furthermore, to provide some insight into the individual-level data and variation, Fig. 2 illustrates how Hb levels change over time for six randomly selected patients from 2012 to 2019.

Given the iterative nature of the estimation procedure of GAMs, an analysing using the entire dataset turned out to be computationally unfeasible. As a compromise, we randomly selected a subset of 30,000 patients based on the upper limit determined through computational experiments. Conducting the full analysis on this sample, which resulted in approximately three hundred thousand records, required 4 days and 8 h of computation on a high-performance computer equipped with 24 cores (Intel Xeon 2.60 GHz) and 120GB of memory, running in parallel.

### Statistical analysis: generalised additive model

In our study, where consecutive measurements of Hb levels for individual patients are taken over time and are inherently correlated, it is imperative to employ a statistical model that effectively accounts for this correlation. Thus, any optimisation function concerning the conditional mean of Hb must appropriately address the dependence on previous Hb values for each patient. Consequently, we utilised the GAMs to analyse the intricate relationship between predictors and the response variable. GAMs offer superior flexibility compared to traditional linear models, accommodating non-linear relationships between predictors and the response variable. This flexibility is particularly vital in medical research, where relationships among variables are complex and non-linear, demanding advanced modelling techniques. Mathematically, GAMs can be expressed as:

$$\:{{\upmu}}_{i}=\:{{\upbeta}}_{0}+{\sum}_{j=1}^{p}{f}_{j}\left({x}_{ij}\right)$$

Here, $\:{{\upmu\:}}_{i}$ represents the expected value of the response variable, Hb. $\:{{\upbeta\:}}_{0}$ denotes the intercept, while $\:{f}_{j}\left({x}_{ij}\right)$ represents smooth functions of the predictor variables $\:{x}_{ij}$, allowing for non-linear relationships. These smooth functions, $\:{f}_{j}$, are selected using the “mgcv” package [19] in R programming [20]. Specifically, “mgcv” employs Generalised Cross-Validation (GCV) or Restricted Maximum Likelihood (REML) to select the optimal smoothing parameter, which determines the extent of smoothness for each function. This selection process ensures that the model captures the complexity of the relationships while avoiding overfitting. The s() function in the “mgcv” package allows each predictor to be specified as a smooth term, with the smoothing amount automatically adjusted based on the data.

Besides, GAMs maintain interpretability, allowing us to understand the effects of individual predictors on the response variable, which is essential for drawing meaningful conclusions from the analysis. The GAMs automatically identify relevant predictors, reducing the need for manual feature selection and streamlining the modelling process. Also, GAMs are robust to outliers and can handle skewed or non-normal distributions of the response variable, leading to more reliable results.

For data processing, we first removed duplicate Hb measurements for each patient taken on the same day and calculated the time since the first Hb measurement for each patient. Additionally, we computed the average Hb value for each patient using all previous Hb measurements up to the prediction time point, excluding any future values. This approach ensures that the model makes predictions based solely on past data, which is essential for maintaining the validity of clinical predictions. The response variable in our model is the Hb level itself, with several predictors used to enhance prediction accuracy. Specifically, time since the first measurement helps to capture temporal variations, while the average Hb value provides insights into a patient’s historical Hb status and allows for meaningful comparisons across individuals. By incorporating these predictors into the model, we aim to enhance our understanding of the factors influencing Hb levels and thereby improve the accuracy of our predictions.

The model included smooth terms for predictors such as average Hb for each patient, time since the first Hb measurement, year of birth, and gender, along with a random effect for patient ID. The inclusion of the random effect for patient ID was intended to capture unobserved individual variability that might not be fully accounted for by the smooth predictor, average Hb. While both the random effect and the average Hb predictor address individual differences, they do so in distinct ways. The smooth term for average Hb captures a patient’s specific Hb profile over time, allowing for non-linear patterns, whereas the random effect accounts for broader unobserved heterogeneity across individuals. Including both terms enabled a comprehensive approach to individual variability, capturing both observed and unobserved components in the relationship between predictors and the response variable, Hb.

We utilised Akaike’s information criterion (AIC) and the Bayesian information criterion (BIC) to select the optimal model structure for predicting Hb levels. For model evaluation, we used two datasets. The first dataset contained all Hb measurements except the most recent one, which was used for model training and parameter estimation. The second dataset included only the most recent Hb measurement for each patient, which allowed us to assess the model’s predictive performance on unseen data. To evaluate the fitted GAMs, we calculated the Mean Squared Prediction Error (MSPE), which measures the average squared difference between predicted and actual Hb values, providing insights into predictive accuracy and the ability to capture relationships between predictors and response variables. Specifically, MSPE is defined as:

$$\:\text{M}\text{S}\text{P}\text{E}\:=\frac{{\sum\:}_{i=1}^{N}{\left({\widehat{y}}_{i}-{y}_{i}\right)}^{2}}{N}$$

Where N represents the number of patients, $\:{\widehat{y}}_{i}$ is the predicted Hb value for an individual $\:i$, and $\:{y}_{i}$ is the actual observed Hb value for the same individual.

Additionally, we computed confidence intervals for the predicted Hb values to assess prediction uncertainty, and the percentage of predictions falling within these intervals was used as a measure of model reliability.

To assess the model’s ability to identify personal ‘out-of-normal’ Hb levels, we established individualised reference ranges for each patient based on historical Hb values, excluding the most recent measurement used for testing. These ranges were defined as mean ± 2.5 times the standard deviation, reflecting an individual’s variability over time. In population-based clinical diagnostics ‘normal ranges’ are defined as mean ± 2 SD, where 2.5% of the values lie above and below the normal range by definition [21].

Our use of ± 2.5 SD for individualised ranges represents a relatively strict criterion for identifying deviations specific to each patient’s historical trends. It ensures that the evaluation focuses on detecting atypical variations from an individual’s own ‘normal,’ rather than broader population-based thresholds.

For evaluating the model’s classification performance, we generated a confusion matrix comparing the predicted and actual classifications of whether the latest Hb value fell within or outside the individualised normal range. Additionally, we calculated metrics such as accuracy, precision, recall (sensitivity), false positive rate (FPR), and F1 score to provide a comprehensive view of the model’s performance, particularly considering the class imbalance between normal and out-of-normal measurements. These metrics help capture the nuances of false positives and false negatives, offering a balanced assessment of the model’s ability to detect deviations from individualised baselines.

To confirm the robustness of our findings, we applied additional SD thresholds around 2 SD (ranging from 1.5 to 2.5 SD) to evaluate model performance across various cutoff values. This analysis allowed us to assess whether the choice of ± 2.5 SD significantly influenced classification results, thereby supporting the stability and generalisability of our selected threshold.

The main function used from the “mgcv” package is “bam” which is performed to fit the GAMs to the large dataset. The model results were visualised using the R-package ggplot2 [22]. For a more detailed explanation of model output metrics, such as estimated degrees of freedom (edf), reference degrees of freedom (ref.df), and F-values, please refer to Appendix A. Appendix A provides further context on the significance of these metrics and their role in evaluating model complexity and significance.

## Result

The results of the GAM analysis for predicting Hb levels are summarised in Table 3. This analysis considers the effects of patient gender, patient age, average Hb, time since the first measurement, and patient ID on Hb levels. The edf for each smooth term illustrates the non-linear relationships between predictors and Hb levels. The model captures a moderately non-linear relationship for patient age, reflecting nuanced changes in Hb across different age ranges (edf = 3.644). The average Hb term allows for slight non-linearity in its relationship with current Hb levels, accommodating individual variations over time (edf = 2.621). The term for time since the first Hb indicates a complex and flexible relationship that captures detailed temporal patterns in Hb levels measurement (edf = 8.854). In contrast, the random effect for patient ID reflects a simpler random intercept approach that captures patient-specific variability without added complexity (edf = 0.090). Together, these smooth terms enhance the model’s ability to capture the non-linear dynamics between Hb levels and each predictor.

The pre-model Intra Class Correlation (ICC) of 0.611 suggests substantial individual differences, which initially justified the inclusion of random effects to account for individual-specific variations. However, the model summary indicated that the contribution of the random effect for patient ID was minimal, with an edf close to zero (edf = 0.090) and a non-significant F-value. This outcome suggests that the smooth term for average Hb, which captures individual Hb profiles over time, accounted for much of the patient-specific variability, thereby reducing the necessity for a separate random intercept. Nevertheless, we retained the random effect to ensure a comprehensive approach to capturing both observed and unobserved individual variability.

An analysis of deviance between the reduced model (excluding random effects) and the full model (including random effects) showed that the inclusion of the random effect did not yield a significant improvement in model performance (F = 0.0884, *p* = .323), with the residual deviance remaining largely unchanged (109877) across both models.

The comprehensive model explained about 65% of the variance in Hb levels. Upon recalculating the ICC for the post-model residuals, a higher ICC value of 0.722 was obtained, reaffirming that individual differences are important. However, the fixed effects, including patient age, average Hb, and time since the first measurement, effectively captured most of the variability in Hb levels, thereby diminishing the impact of including random effects.

The MSPE was 0.48 mmol/L, indicating that the model’s predictions deviate from the true Hb values by an average of 0.48 units within the range of 6 to 16.5 mmol/L. The model’s predictive accuracy was assessed by comparing the predicted Hb values with the actual values from the most recent measurements. Approximately 60.12% of the predictions were within an acceptable error threshold of ± 0.5 mmol/L, and 99.16% of the predictions were within two standard deviations of the mean Hb level.

**Table 3:** Results of the GAM analysis for predicting Hb levels. The table includes estimates for the parametric coefficients, approximate significance of smooth terms, estimated degrees of freedom (edf), reference degrees of freedom (ref.df), F-values, and p-values

Nominal effects				
Independent variables	**Estimate**	**Std. Error**	**t-value**	**p-value**
Intercept	8.6274	0.0033	2545.3	< 0.0001
Patient gender (male)	-0.0053	0.0025	-2.119	0.034

Smooth effects				
Independent variables	**edf**	**ref.df**	**F-value**	**p-value**
Patient age	3.644	4.540	10.8	< 0.0001
Average Hb	2.621	3.322	137,504	< 0.0001
Time since first Hb measurement	8.854	8.992	327.6	< 0.0001
Random effect for patient ID	0.090	29,996	0.00	1

**Fig. 3:**
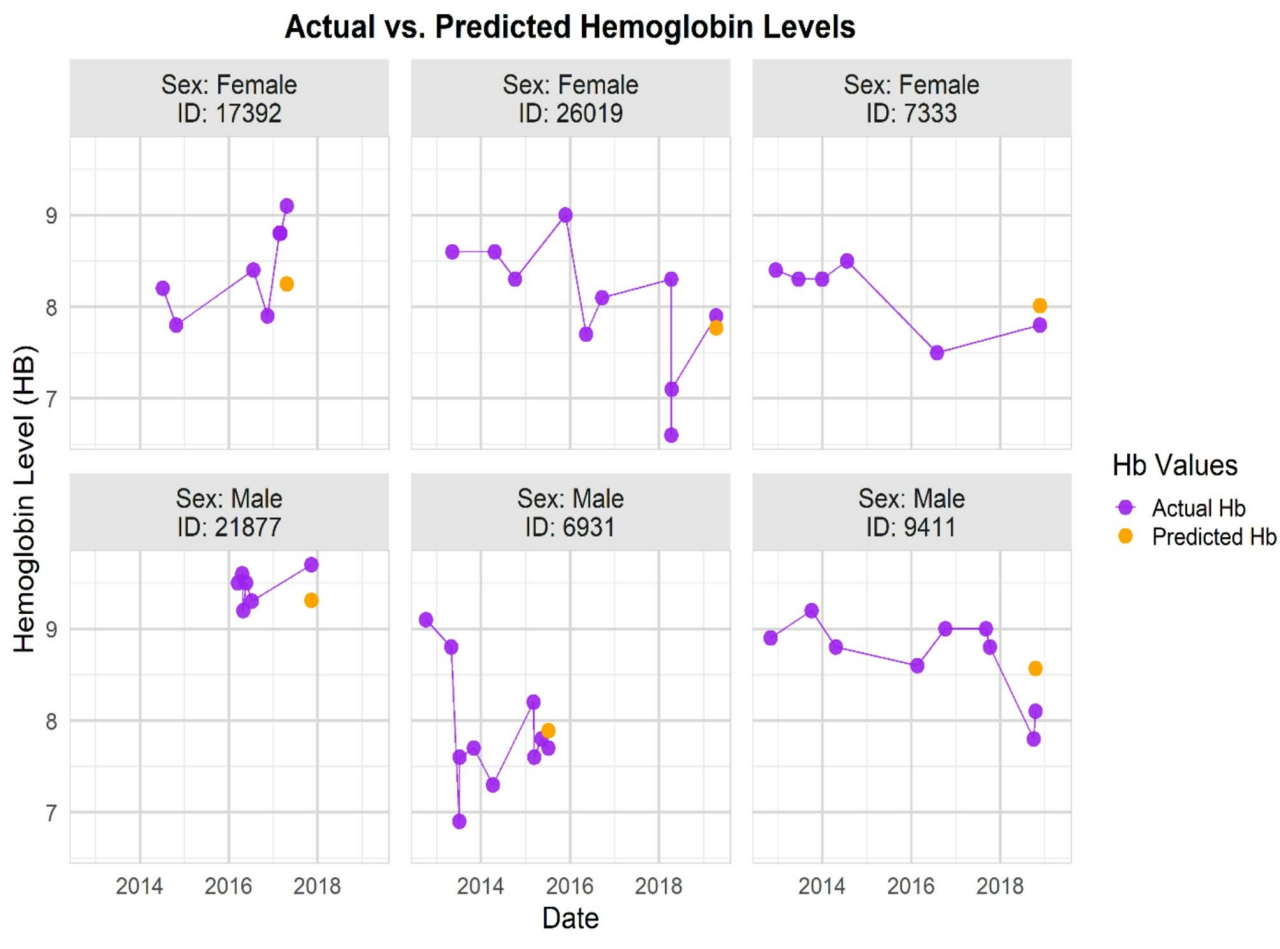
The figure displays actual and predicted Hb levels for six selected patients, with three females shown in the top row and three males in the bottom row. Each subplot represents an individual patient, labelled with their sex and unique ID. Purple points and lines show the historical and most recent actual Hb measurements, while orange points represent the predicted Hb values from the model at the last time point

Moreover, based on the model, Fig. 3 compares selected patients’ actual and predicted Hb levels at the last included time point. The proximity of the predicted Hb values (orange points) to the actual values (purple points) from the most recent measurements demonstrates the accuracy of the model in predicting Hb levels.

Our analysis showed that from the last included time point in the dataset to the prediction point, 371 patients^1^ developed ‘out-of-normal’ Hb measurements, while 26,170 patients^2^ remained within their individualised normal bounds. The prevalence of normal Hb levels in our primary healthcare population was 87.28%, reflecting that most patients did not experience significant Hb deviations during the study period. Given this class imbalance, accuracy alone may not adequately reflect the model’s performance.

To provide a more comprehensive view, we calculated several additional performance metrics. The overall prediction accuracy was approximately 88.47%, indicating how reliably the model predicts whether patients’ Hb levels remain within normal bounds or deviate from them. The model demonstrated a precision of 88.4%, meaning that most positive predictions (i.e., Hb levels predicted as ‘normal’) were correct, reducing unnecessary clinical follow-ups for false positives. The recall (sensitivity) was notably high at 99.95%, demonstrating the model’s success in identifying nearly all true positives. The false positive rate (FPR) was relatively high at 90.28%, indicating a challenge in correctly identifying the relatively small number of out-of-normal cases, which is expected given the class imbalance. The F1 score of 92.9% balanced both precision and recall, indicating that the model effectively manages both true positives and false positives, which is critical in a clinical context.

To summarise the results, we present Table 4 below, which includes the confusion matrix, along with Table 5, which shows the formulas and values for the key metrics.

**Table 4:** Confusion matrix and performance metrics for model prediction of Hb normality

	Predicted normal (true)	Predicted out-of-normal (false)	Total (actual class)
Actual normal (true)	TP^1^: 26,170	FN^2^: 14	TP + FN: 26,184
Actual out-of-normal (false)	FP^3^: 3445	TN^4^: 371	FP + TN: 3816
Total (predicted class)	TP + FP: 29,615	FN + TN: 385	TP + FN + FP + TN: 30,000

^1^ True Positives (TP): Number of cases where both actual and predicted values are within ‘normal’ bounds

^2^ False Negatives (FN): Number of cases where actual values are within ‘normal’ bounds, but predicted values are ‘out-of-normal’ bounds

^3^ False Positives (FP): Number of cases where actual values are ‘out-of-normal’ bounds, but predicted values are within ‘normal’ bounds

^4^ True Negatives (TN): Number of cases where both actual and predicted values are ‘out-of-normal’ bounds

**Table 5:** Performance metrics (%)

Metrics	Values
Prevalence of normal Hb	TP + FN/Total: 87.28%
Prediction accuracy	TP + TN/Total: 88.47%
Precision (positive predictive value)	TP/TP + FP: 88.4%
Recall (sensitive/TPR)	TP/TP + FN: 99.95%
False positive rate (FPR)	FP/FP + TN: 90.28%
F1 score	2 * Precision * Recall / (Precision + Recall): 92.9%

Furthermore, we tested multiple SD thresholds to verify that our model’s performance was not sensitive to the specific choice of ± 2.5 SD. For instance, accuracy ranged from 72.39% at 1.5 SD to 88.47% at 2.5 SD, while sensitivity remained consistently high across all thresholds (99.75–99.95%). A summary of the model’s performance metrics at different cutoff multipliers is provided in Table 6, Appendix B. These results confirm that our model’s classification performance is stable regardless of the specific SD threshold used. By including additional metrics and testing multiple thresholds, we provide a balanced assessment of the model’s strengths and limitations, supporting the robustness of our chosen threshold and remaining in line with the general agreements in clinical diagnostics.

Furthermore, to compare the impact of model complexity on predictive performance in our study, we applied two models to a subset of approximately 100 randomly selected patients. The first model was a simpler version, including smooth terms for average Hb levels, time since the first Hb measurement, year of birth, and gender, along with a random effect for patient ID. This model structure mirrors the model used for the entire dataset. The second model was more sophisticated, incorporating additional predictors and complexities such as interaction effects between the predictors, like different smooth functions for average Hb values by gender and patient-specific effects. Upon comparing the results, we observed that the more sophisticated model exhibited a significantly lower AIC compared to the simpler model, suggesting a better fit in terms of goodness-of-fit versus model complexity. However, despite the lower AIC, the MSPE of the predicted and actual Hb values was higher for the more sophisticated model, indicating that increased complexity did not necessarily translate to better predictive accuracy in this case.

Additionally, to improve predictive accuracy, we refined our analysis by creating a new subset of the dataset. We split the dataset based on gender (female and male) to account for potential differences in Hb levels and to enable gender-specific modelling. Within each gender-based subset, we filtered out patients with Hb measurements outside specific ranges: 6 to 12 mmol/L for females and 7 to 13 mmol/L for males. These ranges are close to the clinical reference values and ensure that only patients with Hb levels within the typical physiological range are included in the analysis. To focus on patients with sufficient longitudinal data for reliable modelling, we excluded individuals with fewer than 3 or more than 20 measurements. Patients with more than 20 measurements often have underlying health conditions that require frequent monitoring, introducing variability related to specific medical issues rather than typical physiological changes. By excluding these individuals, we aimed to focus on a generally healthy population that aligns with our goal of preventive healthcare. Patients included in this subset were typically under regular control in the primary healthcare system, undergoing Hb measurements as part of standard health checks without active clinical symptoms. This approach helped reduce noise, avoid overfitting, and ensured that the model was trained on stable data, leading to improved predictive performance. The resulting MSPE was 0.27 mmol/L for females and 0.25 mmol/L for males, demonstrating enhanced prediction accuracy within this stable population.

Besides, we compared the GAMs with a mixed effects model. The mixed effects model included a random intercept for patient ID, while the GAMs also included smooth functions for predictors such as average Hb level, time since the first measurement, and patient age. We found that the GAM offered enhanced flexibility to capture non-linear relationships in the data, which may be crucial for understanding patient-specific variations in Hb levels. Both models showed almost similar performance metrics, but the additional interpretability provided by the smooth functions in the GAM made it a preferred choice for our analysis. The output of the mixed effects model is provided in Table 7, Appendix C for further comparison.

Although both models achieved similar prediction performance, with comparable MSPE and F1 scores, the GAM’s flexibility in capturing non-linear trends and individual temporal variations offered greater interpretability and insights, which is particularly valuable for personalised healthcare applications.

## Discussion

In this study, we employed the GAMs to predict the last Hb measurement based on previous individual Hb values measured over time. The GAMs effectively accounted for varying baseline levels and trends among different individuals, making them a suitable model for predicting individual Hb trajectories. Our results demonstrated that GAMs can effectively model and predict future individual Hb values using historical data, providing a robust framework for understanding factors influencing Hb dynamics over time.

The GAMs method uses previous observations to make predictions, requiring more historical information than a simple linear regression model. A key advantage of using GAMs in this study is their ability to handle unbalanced time intervals between measurements. This was achieved by defining a variable that considers the days’ interval between each measurement from the first measurement, capturing the dynamic pattern of the time variable within the GAM framework.

Additionally, the proposed GAM model incorporates ‘time since the first measurement’ as a critical time-varying covariate, allowing it to effectively capture temporal dynamics and trends in Hb values (edf = 8.854, *p* < .0001). The use of smooth functions enables the identification of non-linear trends and individualised changes in Hb levels, making the model adept at handling complex time dependencies in the data. These findings highlight the potential of GAMs for accurately modelling and predicting individual Hb levels in clinical settings. Although the random effects did not significantly improve the model fit, the high ICC values before and after the model fitting suggest that individual differences are still relevant.

In this study, we compared models of different levels of complexity to understand how model sophistication impacts predictive performance. The simpler model mirrored the original approach we used for the full dataset and included straightforward predictors such as average Hb, time since the first Hb measurement, year of birth, and gender, with a random effect for patient ID. The more sophisticated model introduced additional smooth terms, allowing for individualised variations such as gender-based and patient-specific interactions with average Hb. Although the more sophisticated model showed a lower AIC, which suggested it might have a better balance between goodness of fit and complexity, the higher MSPE indicated that this complexity did not yield better predictions. This suggests that in clinical settings, an overly sophisticated model may overfit individual nuances that do not generalise well to new data, thus impacting the practical utility of the model. Balancing model complexity and prediction accuracy is crucial for clinical applications, where interpretable and consistent predictions can make a significant difference in patient care.

Besides, to refine our analysis and improve predictive accuracy, we created gender-based subsets within the dataset. By recognising potential gender discrepancies in Hb levels and adjusting our analyses accordingly, we aimed to achieve more precise and relevant findings. By targeting specific patient populations with consistent Hb levels and sufficient longitudinal data, the analysis provided valuable insights into the factors influencing Hb dynamics and enabled more accurate predictions of future Hb levels.

One of the key potential applications of the individualised Hb prediction model is the ability to facilitate early detection and intervention, ultimately improving patient outcomes and enhancing healthcare efficiency in preventive medicine. By continuously monitoring a patient’s actual Hb measurements against their personalised predicted values, we can identify significant discrepancies that may warrant medical attention. For instance, if a patient’s observed Hb level deviates substantially from their predicted value based on historical trends, it could signal a potential underlying health issue or the need for further investigation or advice.

The proposed model requires a minimum of three prior Hb measurements to make reliable predictions. This threshold ensures that sufficient data is available to establish a stable individual trend for each patient. The inclusion of time since the first measurement as a predictor also enables the model to effectively account for gradual shifts in Hb levels, ensuring that changes in individual health status are captured in a meaningful way. For clinical use, this makes the model particularly suitable for patients undergoing regular monitoring, allowing for accurate and personalised predictions that reflect both gradual and dynamic changes in Hb levels. In such cases, our improved model could automatically alert medical professionals, prompting them to intervene proactively and potentially prevent the development of more severe conditions. Furthermore, the individualised nature of our approach allows for the use of personalised reference ranges or thresholds, rather than relying on population-based references. This could enable more sensitive and specific monitoring, as deviations from an individual’s typical range may be more indicative of potential issues than deviations from a broad population range.

Interestingly, our findings align closely with recent high-impact research demonstrating that haematological setpoints, including those for Hb, can be reliably estimated with as few as four independent tests. This convergence of results not only validates our approach of using individualised baselines for Hb level predictions but also suggests a potential reshaping of preventive medicine through personalised reference ranges [15].

While our current study focuses primarily on predictive performance, future research could explore the development of a robust monitoring system that integrates our prediction model with automated alerting mechanisms. Such a system could continuously evaluate incoming Hb measurements against personalised predictions and thresholds, triggering alerts when significant discrepancies are detected. This would not only facilitate early intervention but also reduce the burden on medical professionals by automating the monitoring process and highlighting only the most relevant cases for review.

Although our prediction model provides valuable insights, there are areas in which it can be improved. Further research is needed to enhance its efficacy, by delving deeper into uncovering novel predictors that influence Hb dynamics, exploring innovative methodologies to improve predictive accuracy, and optimising the timing of interventions based on predictive insights. Future investigations could explore alternative probabilistic approaches for defining individualised reference ranges, such as percentile-based thresholds, Bayesian credible intervals, or Gaussian Process models, which may offer different perspectives on patient-specific Hb variations. Additionally, future studies could explore additional factors that may uniquely influence Hb levels, such as weight, dietary habits, physical activity, environmental exposures, and medical history. Furthermore, it is important to carefully consider the timing of future Hb measurements in order to reduce the need for patient hospital visits while increasing opportunities for preventive interventions.

In sum, our study highlights the potential of predictive modelling in preventive healthcare, particularly in predicting individual Hb levels to improve patient outcomes and healthcare efficiency. While our current model shows promise, future research is essential to refine predictive accuracy and explore novel predictors. By utilising advanced modelling techniques and incorporating multiple factors, we can improve preventive medicine and optimise patient care.

## Conclusion

This research in preventive medicine has potential practical implications, especially in preventing unnecessary blood draws and identifying unexpected discrepancies between predicted and measured values. Our model shows promise in predicting Hb levels, which could have a significant impact on clinical care. In addition, this work also contributes to the use of individual, instead of population-based, normal ranges as the next step in personalised medicine. By improving patient outcomes and enhancing healthcare efficiency, this study highlights the potential of predictive modelling for blood values in preventive healthcare.

## Data Availability

The datasets used and analysed during the current study are not publicly available but are available from the corresponding author on reasonable request.

## Appendix A

### Model metrics and interpretation

The output of the GAMs includes several important metrics that are critical for assessing the complexity and fit of the model. The estimated degrees of freedom (edf) provide insight into the complexity of each smooth function within the model. A value of 1 indicates a linear relationship, while higher values suggest that the smooth term captures a more complex, non-linear pattern. The reference degrees of freedom (ref.df) represent the theoretical degrees of freedom used in hypothesis testing, providing a measure of the statistical reliability of the smooth term. The F-value indicates the ratio of variance explained by the smooth term relative to the residual variance and is used in an ANOVA-like test to assess the significance of the smooth term. Together, these metrics help evaluate the contribution and significance of each smooth term in describing the underlying data patterns effectively.

## Appendix B

### Summary of the model’s performance metrics at different cutoff multipliers

**Table 6:** Overview of model performance across cutoff multipliers (%)

Cutoff multiplier	Sensitivity	Specificity	Prediction accuracy
1.5	99.75%	5.44%	72.39%
1.7	99.85%	6.01%	77.10%
1.9	99.90%	6.79%	80.80%
2.1	99.93%	7.52%	83.42%
2.3	99.94%	8.72%	86.73%
2.5	99.95%	9.72%	88.47%

This result demonstrates that the model’s performance remains consistent and robust across different SD thresholds, with sensitivity staying consistently high while specificity and accuracy improve as the cutoff multiplier increases.

## Appendix C

### Summary of linear mixed effects model for predicting Hb levels

**Table 7:** Results for the linear mixed effects model predicting Hb levels

Predictor	B	SE	t	95% CI	*p*-value
Intercept	0.849	0.128	6.61	(0.599, 1.099)	$$\:{<.001}^{***}$$
Patient gender (male)	-0.00561	0.00247	-2.27	(-0.0105, -0.0007)	$\:{.023}^{*}$
Patient age	-0.00042	0.00007	-6.38	(0.00055, -0.00028)	$$\:{<.001}^{***}$$
Average Hb	1.005	0.001	687.67	(1.003, 1.007)	$$\:{<.001}^{***}$$
Time since first Hb measurement	-0.00008	0.000002	-47.29	(-0.00008, -0.00007)	$$\:{<.001}^{***}$$

A mixed effect model is run for the same data set and these results are compared with our GAMs. The mixed effects model included fixed and random effects, with the variance associated with patient ID estimated at nearly zero, indicating minimal individual variability after accounting for fixed effects. The residual variance was 0.3867, with a marginal R² of 0.649, suggesting that 64.9% of the Hb level variance was explained by the fixed effects. Key predictors such as average Hb (B = 1.005, *p* < .001) and time since first measurement (B = -0.00008, *p* < .001) were significant, showing the impact of historical averages and slight declines over time. Sex (male) also had a minor effect (B = -0.00561, *p* = .023).

Both models showed comparable predictive accuracy, with an MSPE of 0.48 mmol/L. Individualised normal ranges were defined using mean ± 2.5 standard deviations, and both models achieved high precision and recall, with F1 scores of 94.33% (GAMs) and 94.37% (mixed effects model). High recall indicated effective identification of “normal” Hb levels, but both models had high false positive rates (90.29% for the GAMs, 90.03% for the mixed effects model).

Although the prediction performance was similar, the GAMs offered better flexibility and interpretability through their use of smooth functions, providing more insight into non-linear trends in Hb levels and capturing patient-specific temporal changes, which is beneficial for personalised healthcare.

## Footnotes

1 These are cases where the model correctly predicted ‘out-of-normal’ Hb levels (actual ‘out-of-normal’ and predicted ‘out-of-normal’).

2 These are cases where the model correctly predicted ‘normal’ Hb levels (actual ‘normal’ and predicted ‘normal’).

## Abbreviations

Hb: Hemoglobin
GAMs: Generalised Additive Models
GCV: Generalised Cross-Validation
REML: Restricted Maximum Likelihood
MSPE: Mean Squared Prediction Error
ETZ: Elisabeth-Tweesteden Hospital
M: Mean
SD: Standard Deviation
AIC: Akaike’s information criterion
BIC: Bayesian information criterion
ICC: Intra Class Correlation
edf: estimated degrees of freedom
ref.df: reference degrees of freedom
